# An emerging knowledge exchange framework: Leadership insight into a key capacity-building impact in a large urban, trauma-informed initiative supporting resiliency and promoting equity

**DOI:** 10.1186/s12889-025-22268-4

**Published:** 2025-05-13

**Authors:** Amy E. Lansing, Natalie J. Romero, Elizabeth Siantz, Kimberly Center, Todd Gilmer

**Affiliations:** 1 Department of Psychiatry, University of California, San Diego, La Jolla, CA USA; 2 Department of Sociology, San Diego State University, San Diego, CA USA; 3 School of Social Work, San Diego State University, San Diego, CA USA; 4 College of Social Work, University of Utah, Salt Lake City, UT, USA; 5 Herbert Wertheim School of Public Health and Human Longevity Science, University of California, San Diego, La Jolla, CA USA

**Keywords:** Community capacity building, Collective impact, Trauma informed practices, Equity, Knowledge exchange, dissemination, transfer and translation, Health disparities, Adverse childhood experiences, Trust-building

## Abstract

**Background:**

Community capacity-building is the cornerstone of many public health initiatives, with increasing attention paid to community engagement, collective impact, and trauma-informed practices designed to support resiliency and promote health equity. Large-scale capacity-building projects proliferated during the global pandemic, highlighting the need for practical guidance and steps for efficiently responding to changing community needs and effectively communicating information across partnership and community members. The present qualitative longitudinal study provides a pragmatic framework for trauma-informed knowledge exchange across stakeholders in a large urban collective impact, capacity-building initiative designed to establish partnerships that engage community members, promote equity through tailored referrals and resource access, and address community needs and aspirations.

**Methods:**

Interviews were conducted with the leads of nine agencies funded to implement regionally responsive strategies addressing adverse childhood experience-driven health needs among their diverse subcommunities, while offsetting the impact of trauma, building capacities and improving resource access. We aimed to capture (1) the socioecological context of traumatic experiences and health barriers that propelled agencies to participate in a trauma-informed initiative; (2) agency leads’ vision for community health; and (3) unfolding approaches to the initiatives’ complex work, spanning pre-pandemic community needs and pandemic era challenges.

**Results:**

Agency leads’ vision for healthy communities emerged from the strengths, adversity-driven challenges and health barriers of their communities; while focusing on relationship-building, trust-based engagement and equitable access to trauma-informed resources through knowledge exchange. Results support reflection-based learning practices that are characterized by a flexible mindset and action-oriented adaptability. Mechanisms that power multi-directional knowledge exchange included creative partnering; frameworks and trainings that address partnership and community needs; and actionable skill-building. Incorporating community members directly into the initiative’s work exemplified the vision of an informed/resourced community, relationship-based engagement, use of adaptive practices and creative partnering. Lived experience staff provided a credibility bridge facilitating knowledge exchange between community and partnership members and creating power-sharing opportunities.

**Conclusions:**

Engagement in public health initiatives is essential for community well-being and responsive public health initiatives. These data provide an emerging framework for thoughtful engagement and knowledge exchange among partnership and community members, while highlighting knowledge exchange as a key impact for outcome consideration.

**Supplementary Information:**

The online version contains supplementary material available at 10.1186/s12889-025-22268-4.

## Background

Over the last 25 years, adverse childhood experiences (ACEs) have been recognized as significant social determinants of health occurring at high-rates across the world and incurring substantial costs – as high as 3–6% of a given country’s annual gross domestic product [1–7]. While ACEs traditionally focused on child maltreatment and family distress indicators such as incarceration, mental illness and substance use, large-scale, chronic and complex adversity exposures such as poverty and racism are similarly linked to health outcomes and are increasingly captured under an expanded ACE umbrella [8–13]. ACEs are associated with higher rates of morbidity and mortality, and increased probabilities of victimization, perpetration and engagement in health-risk behaviors [12, 14]. Higher prevalence of ACEs are observed globally among racial/ethnic minorities and individuals experiencing low-income [15, 16]. While health agencies and institutions around the world recognize the public health costs and lost productivity associated with ACEs [7], the use of trauma-informed practices to mitigate and address the negative consequences of ACEs is relatively recent, arising initially within mental health and substance use treatment contexts, followed by implementation in other systems (e.g., education, juvenile justice, child welfare), and more recently, entire communities [17–22]. 

Efforts to implement trauma-informed practices at the community level aim to promote awareness and understanding about the (a) impact of trauma, (b) signs and symptoms of trauma exposure, and (c) importance of actions that increase emotional and physical safety while avoiding re-traumatization by providing information to the general public and specific stakeholders such as community leaders, parents and school staff [23, 24]. Community-wide efforts are more universal than trauma-informed direct service delivery efforts, often focusing on risk prevention related to violence, mental illness and substance use; public health promotion, including healthy development; and capacity building focused on skills, knowledge and resources [25–27]. While not typically treatment-focused, these efforts may include connecting community members with needed resources, promoting positive community relationships between the public and agencies or systems delivering services, and enhancing the health promotion capacity of service systems themselves [28, 29]. Providing evidence-based information directly to all community members – including the communities of health providers and systems of care - has the potential to enhance overall community resilience.

While resiliency has many connotations, at the community-level disaster preparedness and recovery is often the focus, with particular attention in recent decades to the impact of climate change [30–34]. Nine converging elements of community resiliency have been identified, capturing local knowledge, community networks/relationships, communication, health, governance/leadership, resources, economic investment, preparedness and mental outlook [34]. For the purpose of broad trauma-informed efforts that may consider individual and community levels of resiliency simultaneously or interchangeably, resiliency can also be operationalized as the safety and well-being of community members and the capacity to mitigate the negative impacts of trauma through healthy adaptation to, and recovery from, the changes and challenges that whole communities, and/or the individuals within those communities, experience [35]. The Community Resiliency Model recognizes this intertwining of community-level and individual-level resiliency [36]. Specifically, community resiliency relies not only on the strengths of the overall community to respond to environmental and societal challenges, but is also deeply rooted in the neurobiology of trauma, acknowledging the importance of its community members’ capacity to self-regulate when experiencing stress.

Collective impact approaches to capacity-building that enhance resiliency aim to develop cross-sector collaborative partnerships with government agencies, community-based organizations and community members that coordinate efforts to increase equity and promote systemic change [37, 38]. While collective impact approaches are, at least in part, a response to public resource scarcities and challenges in service delivery, capacity-building entails the formation of enduring knowledge, resources and skills that can be sustained in the absence of external funding [20, 39–41]. Of note, there is also no consensus on a single definition for capacity-building. This is likely because of its interdisciplinary nature, which encompasses different capacities (e.g., social, health, economic), strategies to promote capacities (e.g., asset development, training, peer networking, resource-identification), and types of “communities” (e.g., lay public, health service providers, organizations, governments) [29, 42, 43]. Despite this lack of consensus, three unifying elements of capacity-building have been identified indicating that it is a “process-oriented” approach; captures a range of capabilities, skills and dimensions; and includes a specific rationale to address needs and/or achieve desirable outcomes [44]. Potential for social change is maximized when capacities are built across sectors, engagement is mutual, decision-making is shared, efforts are coordinated, and communication is consistent [37, 45–48]. 

Capacity-building is one effective way to help communities adapt to change, successfully navigate and recover from challenges or crises (i.e., resiliency) at the individual-level of community members and at the community-level, including how skilled, knowledgeable and responsive partnerships are in supporting the communities they serve as well as their own staff [49–54]. Capacity-building efforts have also been linked to community well-being [55, 56]. Although well-being has been operationalized in various ways, we approach well-being as a broad, holistic construct related to health, quality of life and flourishing that encompasses cultural, economic, environmental, political and social dimensions. Well-being also dynamically occurs both objectively and subjectively at the scale of individuals, communities and populations across physical and geographical levels (e.g., neighborhoods, regions) [41, 54, 57–61]. 

An important process-oriented approach to building capacities that support well-being and resiliency is knowledge exchange, which can enhance the effectiveness of collective impact initiatives through non-hierarchical cross-pollination of ideas and sharing of information among stakeholders [62, 63]. Importantly, knowledge exchange supports many of the identified core elements of resiliency [34], with particular attention to knowledge relevant to health outcomes and available resources; community networks and relationships; and communication. Further, knowledge exchange encompasses scientifically established practices to train providers and/or empower community members; practical information about community needs, resource adequacy and resource availability; innovative ideas; and tacit knowledge gained through community members’ lived experiences alongside providers’ experiences, bridging scientifically-derived findings and real-world client needs [63–68]. Knowledge exchange has the potential to be a democratizing process that places information in the hands of the very individuals who have the capacity, skills, and resources to take action (i.e., knowledge-to-action). In principle, it also values both scientific (codified content) and lay community (context-driven tacit knowledge, or ‘know-how’, drawn pragmatically from experience) expertise from those whose lived experiences and dedication are often primarily responsible for the successful uptake in public health initiatives [69–74]. 

Notably, a plethora of interrelated terms have been used to describe the flow of health-related information (e.g., knowledge transfer, translation, diffusion, dissemination), with definitions traditionally describing a linear, unidirectional process where knowledge is generated by scientific professionals and then distributed by them to providers and the public [42, 62, 75]. However, researchers increasingly recognize that knowledge exchange is a nonlinear process requiring engagement and multi-directional communication among stakeholders [76–78]. In this study, we embrace the more inclusive Canadian Health Services Research Foundation’s definition of knowledge exchange as an interactive flow of information that places knowledge in the hands of stakeholders while allowing feedback about “knowledge-user” needs and emphasizing mutual learning within a non-linear relational model consistent with intersecting public-private ecosystems [66, 75, 79]. This aligns with Greenhalgh and Wieringa’s (2011) call to use more inclusive terms when describing the value of “collectively negotiated” knowledge [80]. These definitions place an emphasis on collaborative relationships, equity-based approaches to the co-production of knowledge, and the coordinated engagement necessary for maximum impact and the sustainability of public health efforts [81–83]. 

An important limitation in the public health knowledge exchange literature is that most data reflect traditional academic community partnerships, but less is known about the real-world application of knowledge exchange strategies, particularly when researchers are not directly disseminating information [84]. Real-world initiatives present important opportunities to understand dynamic culture change, in vivo problem-solving in the face of obstacles, and organically emerging steps and solutions for successful, collaborative partnerships supporting public health in ways more palatable to the broader public. Further, real-world initiatives stand to benefit from a broader conceptualization of knowledge exchange that recognizes the importance of tacit knowledge and lived experience communicated by members of the public to the agencies and organizations ostensibly designed to address their needs.

The present study provides insight into a collective impact initiative designed to build capacities through a trauma-informed lens that supports resiliency. We captured the (1) socioecological context of trauma experiences and barriers to well-being that propelled agencies to participate in a trauma-informed initiative; (2) agency leads’ vision for health and well-being in their communities; (3) relational focus embraced by the partnerships (e.g., engagement, relationship-building); and (4) unfolding approaches to the initiatives’ complex work that spanned pre-pandemic and pandemic era challenges. Our goal is to provide a framework from a real-world initiative that is relevant to a range of public health initiatives, facilitates actionable practices and mechanisms underlying capacity-building and knowledge exchange, and supports knowledge exchange as a key impact relevant to a wide-range of initiatives.

## Methods

### Project setting and overview

Recognizing that expanded ACEs are important social determinants of health, the Los Angeles County Department of Mental Health (DMH) designed Innovations 2 (INN2) as a learning-based initiative that funded lead agencies to form novel partnerships and build capacities around trauma-informed practices with, and for, stakeholders including social service agencies, community partners, and community members, while addressing specific regional community goals and needs. All participating agencies saw gaps and/or opportunities related to trauma-informed practices based on their existing work in the communities they served, with eight agencies noting that INN2 was a match or “marriage” with the goals and vision they were already collaboratively working on with their communities. Six of the nine agencies incorporated specific listening sessions, focus groups and/or asset mapping (i.e., collaboratively identifying, describing and geographically visualizing existing resources and assets) with community leaders, neighborhood associations and other groups represented within their community (e.g., Cambodian and Latinx groups, Alcoholics Anonymous).

Exchanging knowledge about the adversity that community members faced and trauma-informed practices both internally within agency/partnership and across partnerships, and externally with the community, was viewed as an initiative-wide form of capacity-building that would raise overall awareness about potential needs, reduce barriers to service access, and support resiliency. Trauma-informed practices were based on principles established by the Center for Disease Control and the Substance Abuse and Mental Health Services Administration: (1) Safety; (2) Trustworthiness and Transparency; (3) Peer Support; (4) Collaboration and Mutuality; (5) Empowerment, Voice and Choice; and (6) Cultural Competency [21]. 

INN2 met established criteria for collective impact to address trauma in the community: a common agenda, shared measurement, mutually reinforcing activities, continuous communication, and infrastructure support across partnerships [85]. The initiative was designed to:

- engage a wide cross-section of community members who may benefit from, but are not typically engaged with, mental health services;
- reduce disenfranchisement by increasing community members’ access to resources;
- increase lead agency capacity by widening the scope of partnering agencies, programs and organizations available to address community needs that fell outside of direct delivery of mental health services; and
- enhance stakeholder (agencies, partnerships, community) capacities through knowledge exchange.

Together these four facets constitute “the work” of the initiative. Knowledge exchange included internal monthly partnership meetings, as well as quarterly initiative-wide Learning Sessions that provided space for information flow across all partnership members and strategies. These sessions encompassed “lessons learned” from each partnership; assessment reports from the university-based evaluation team; input from DMH; and expert-led, evidence-based workshops.

Knowledge exchange also occurred through partnership- and community-based trainings in trauma-informed practices (See Supplement Table 1), and a range of community-based engagement opportunities (Supplement Table 2, Supplement Fig. 1). Knowledge exchange was amplified between partnerships and their communities via staff with lived experience: Peer Navigators (transition age youth who were age-mates to youth populations served in some of INN2’s strategies), Promatores de Salud (Spanish-speaking health navigators), and Community Ambassadors (lay mental health workers living in the communities they support). Consistent with emerging data, these staff provided a credibility bridge between partnerships and their community members and facilitated more effective exchanges of public health information to community members, and community members’ tacit and experiential knowledge to agencies and partnerships [41, 54, 57, 86]. Staff with lived experience in the communities they served were also key players in linking community members with relevant service referrals, particularly in historically underserved cultural and linguistic communities. Lived experience was fluid throughout the partnerships and over time, with Community Ambassadors experiencing similar struggles to the community members they served (e.g., inadequately housed), currently living in the community they served and/or previous residents of the community [86]. 

Alongside the broader work of the initiative (e.g., trauma-informed knowledge exchange), the nine partnerships were funded to implement one or more of seven trauma-informed community capacity-building strategies that focused on specific demographic groups (e.g., transition age youth). Strategies either focused more directly on preventing ACEs (e.g., skill-building for parents with children under the age of five) or addressing consequences often associated with ACEs (e.g., homelessness). Agencies chose capacity-building strategies unique to their subcommunity with specific, supportive approaches to build skills, increase social connectedness, promote resource access, and reduce the impact of adversity (details previously published) [87–90]. All strategies included outreach and engagement efforts while providing links to resources and services for their community members such as knowledge exchange about trauma-informed practices to teachers and parents, and facilitating societal re-entry among previously incarcerated individuals.

Each partnership reflected a specific geographic region within Los Angeles County, the most populous county in the US with 9.7 million inhabitants [91]. The county is racially and ethnically diverse, with Latinx community members accounting for nearly half the population (49.1%), followed by White non-Latinx (25.2%), Asian (15.1%), Black (7.9%), Multiracial (2.3%), Native Hawaiian and Other Pacific Islander (0.2%), and American Indian/Alaska Native (0.2%). Over 56% of households speak a language other than English in their homes, and 14.2% of the population lives in poverty [92–95]. 

**Fig. 1:**
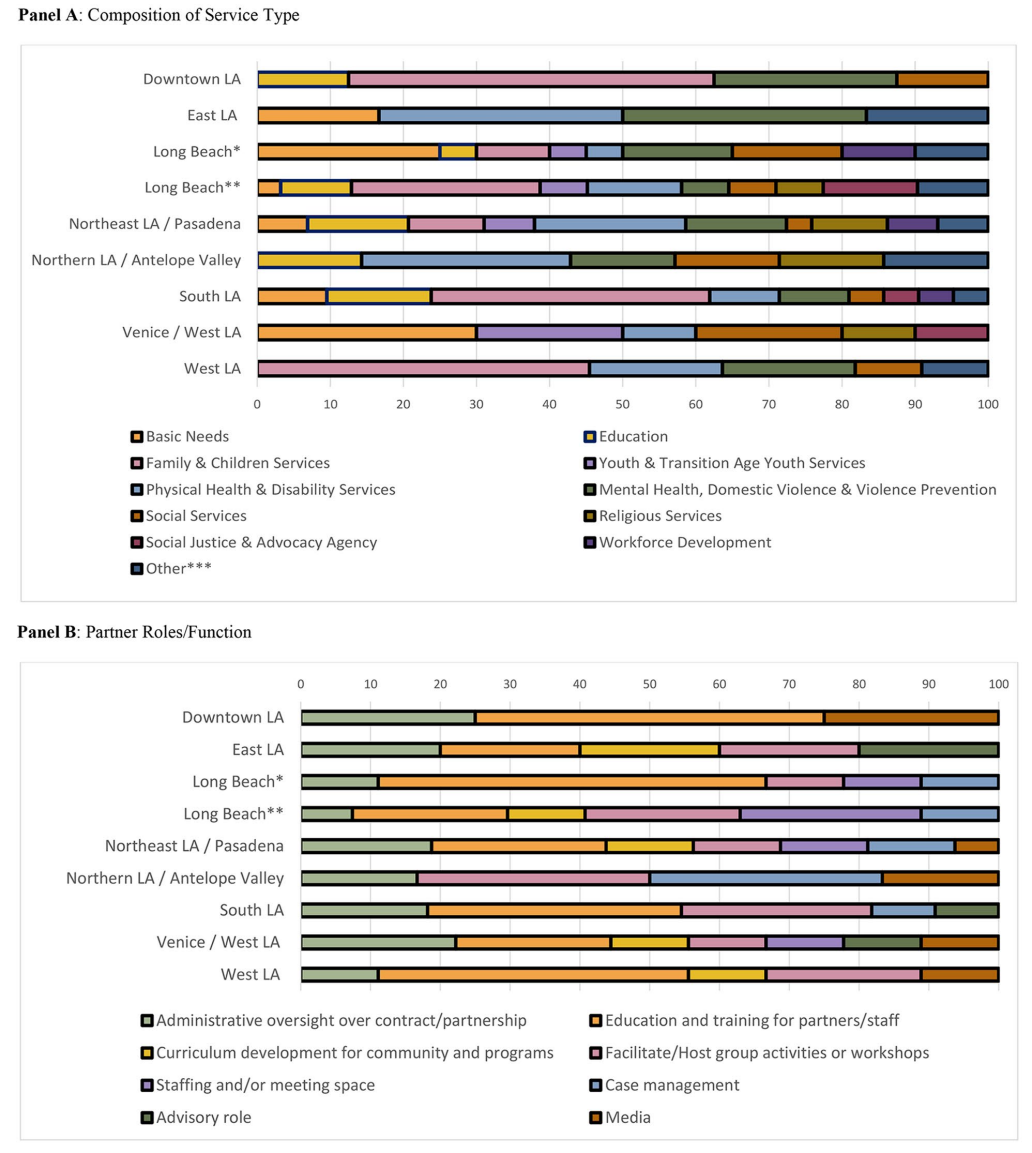
Service types and roles of participating agencies among nine INN2 partnership networks. *Note.* The entire region of Long Beach was served by two partnerships, representing distinct strategies for specific populations. *City of Long Beach partnership serving Families and Young Children; **City of Long Beach partnership serving Transition Age Youth and Adults. ***“Other” service types in Panel A include legal and technological services

Partnering organizations were chosen within each region and strategy to improve resource access, address community needs, and support community health and well-being. Partnerships serviced geographic regions of different sizes, implemented different combinations of strategies and evolved over time to adjust for community needs. Figure 1 provides a mid-initiative view of INN2 partnerships, highlighting both similarities and nuance across the partnerships serving different communities. Panel A illustrates the type of organizations comprising each partnership. While all partnerships included organizations providing resources, linkages, direct services, and community engagement and outreach; Panel B highlights more distinctive functions among organizations participating in the nine partnerships. Many partners also served multiple roles and functions, but only each partner’s primary role is reflected.

### Ethics approval and consent to participate

Methods were carried out under relevant guidelines/regulations and reviewed and approved by the University of California, San Diego Institutional Review Board (UCSD IRB #201892X). Data were collected as part of a program evaluation supporting multiple partnerships in building community capacity to access resources and offset the impact of community trauma. UCSD’s IRB determined the secondary use of the data for research purposes presented no more than minimal risk to human subjects. The study qualified for expedited review. Informed consent was waived by UCSD’s IRB as risk was deemed minimal.

### Study sample

Thirty-one interviews were conducted with the agency leads from each partnership. Agency leads were selected for interview because they were familiar with the original grant application; were key informants knowledgeable about how their agency, partnership, and strategies evolved over time; directly interacted with community leaders and members; were instrumental in decision-making; established the tone of their collaborations; and were critical to upholding to the vision and mission of their partnerships. If a partnership had multiple leads representing different strategies, separate interviews were conducted. Demographics for the agency leads were previously reported by year [88], with 10–11 interviews conducted annually over three years. Across the three waves, agency lead participants were largely female (80%) and racially/ethnically diverse (29% Black; 36% Latinx; 23% non-Latinx White, and 10% each Asian and Biracial/multi-ethnic), with one to two partnerships having two leads (see Lansing et al., 2023 for annual breakdown) [88]. 

### Interview guide development and data collection waves

Interview questions were designed to provide opportunities to discuss INN2 approaches, frameworks, and strategies; reflect on challenges and lessons learned; integrate insights across partnerships reflecting different strategy compositions; and track how the initiative unfolded as circumstances changed (e.g., onset of pandemic). Three waves of interviews were conducted. Baseline interviews occurred pre-pandemic, midpoint interviews occurred during the height of the pandemic, and final interviews occurred post-vaccination availability as restrictions were being lifted, but before the World Health Organization declared the end of the Public Health Emergency of International Concern [96]. Interview guides were developed by one evaluation team member (a clinical psychologist), based on emerging circumstances (e.g., pandemic), and meeting discussions including: Learning Sessions held across-partnerships, the research team and DMH; combined within-partnership and community member meetings; and DMH meetings with individual partnerships. Relevant prompts and follow-up questions facilitated consistent acquisition of information across interviewers. Questions and prompts were collaboratively refined by the evaluation team including staff and faculty trained in clinical psychology, health policy, social work, program evaluation and public health. All interviews were conducted by the same MA and PhD trained members of the evaluation team across all interview waves. Interviewers debriefed after their first interview in each of the three waves to facilitate question refinement and query alignment, as needed. Example questions were previously published [88, 89]. 

Ten in-person baseline interviews occurred in August and September of 2019. Questions captured (1) implementation, including how agency leads adapted their strategies and reevaluated partners following delayed funding; (2) agency and partnership communication networks to supplement quantitative social network data; [89, 90] (3) pressing community needs, specific trauma/adversity experiences and perceived obstacles to health in their respective communities prior to the initiative’s implementation; (4) nuance in language (e.g., what “trauma” means to different stakeholders); and (5) agency leads’ vision and aspirational goals for their community. Example questions included:

- *When you think about “trauma” or “ACEs” in your community*,* what types of experiences come to mind?*
- *When the grant application was written*,* what did your agency perceive as your community’s biggest challenge for overall well-being and health*?
- *What is the main thing you are trying to achieve as a community*?

In November and December of 2020, eleven midpoint interviews occurred via Zoom, and addressed how the partnerships responded to shutdown orders, changes in economic and resource availability, and increased food insecurity/residential instability due to COVID-19. Year two was also marked by discrimination-related civil unrest in the US. Questions queried: (1) emergent themes from the baseline interview (e.g., relationship- and trust-building), (2) the trauma-informed lens for capacity-building; (3) collaborative work with community members to address urgent pandemic- and civil unrest-related needs; and (4) observed impacts and sustainability considerations (e.g., sharing goals, power and decision-making directly with the community, hiring more community members with lived experience). Example questions include:

- *What are the primary barriers to addressing the impact of adversity and trauma in your community?*
- *What does the community most need to support their recovery from the pandemic based on your observations and meetings with your community members*,* participants and partners*?
- *What do you think will be the most important outcome of this initiative for your community and its members?*

Ten final interviews were conducted in November 2021, via Zoom. Reflection-based questions focused on the impact of (1) learning-based cultures; (2) ACEs and trauma-informed practices; (3) strengths-based frameworks; and (4) directly involving community members in the initiative’s work, as this became an increasingly formalized approach over the course of the initiative. While all partnerships included lived experience staff when INN2 began, funding was allocated in 2020 to support these community member staff under a formalized Community Ambassador Network umbrella (see Siantz et al., 2024 for additional details on this network) [86]. Example questions include:

- *How has the concept of resiliency in general*,* or the Community Resiliency Model specifically*,* changed your thinking about the community and/or how you connect with your community*?
- *Other than INN2-based training like Community Resiliency Model*,* Trauma-informed practices and Mental Health First Aid*,* what kinds of support or training have you found most useful for frontline staff who have lived experience*?
- *How has community involvement*,* the incorporation of frontline staff who have lived experience*,* and the mutual exchange of ideas and knowledge*,* changed your thinking about INN2’s work*?

### Data analysis

Our qualitative coding practices were based on Grounded Theory [97–99]. Data collection and coding co-occurred, with coding refined in an ongoing manner across the three interview waves and a core coding team of two to four BS- and PhD-level coders. For each wave of coding and all aspects of coding development, at least one coder was a staff researcher and at least one was a co-investigator who also conducted approximately one-third of all interviews. During the baseline wave, two co-investigators were directly involved in coding. As originally noted by Glaser (1978), it is particularly important for theoretical development and coding that invested researchers actively participate in coding and not relegate all coding tasks to staff [100]. Other evaluation team members provided input over the coding waves, as new questions were developed for subsequent interviews and during final analyses for manuscripts. Grounded Theory coding is an inductive, rather than hypothesis-driven, process that evolves based on organically unfolding stories that emerge from the accumulation and repetitive analysis of data [101]. However, given the semi-structured nature of our interviews, code development naturally included mixed deductive (initial codes linked to specific areas of inquiry) and inductive (theories and frameworks that emerged from the data over time) iterations.

Comprehensive *First Cycle* Coding (e.g., in vivo, descriptive and process coding) of all interviews followed open coding of > 35% of all full transcript interviews per interview wave. *Second Cycle* coding included focused (thematic and conceptual analysis), axial (dimensional) and theoretical coding iterations. Memo codes and peer debriefing were used throughout coding efforts to align and synthesize input from coders. *Third Cycle* coding (reflections on the substantive theoretical, methodological and framework development) and negative case analyses (identifying and discussing contradictory data) occurred between the primary coder and PhD-level supervisor who was involved in coding all interview waves. Themes were developed through review of quotes and codes for each agency lead across questions within each wave, and across time periods, as well as between agency leads within and across the three waves. Our coding process has been extensively detailed in prior publications [88–90]. 

### Positionality statement

Our interdisciplinary team is comprised of predominately female, non-Latinx white and Latina (e.g., Mexican Americans, first generation Salvadoran American) professorial and graduate student community-based researchers who are studying efforts by nonprofit organizations to build capacity in communities that are affected by trauma. Team members also have expertise on the impact of developmental trauma and qualitative and quantitative data analysis. We are largely middle class, cis-gendered and monolingual English speakers residing in Southern California. One member is non-binary, two are bilingual and other represented racial/ethnic groups include Filipino and Indian. One member of the team lived in, and has multi-generational ties to, Los Angeles, and another has experienced both residential instability and food insecurity in two countries. All members of the research team were actively engaged with partnership members in Los Angeles. We recognize the privilege associated with our backgrounds and seek to utilize these identities to advocate for those who are less advantaged.

## Results

**Fig. 2:**
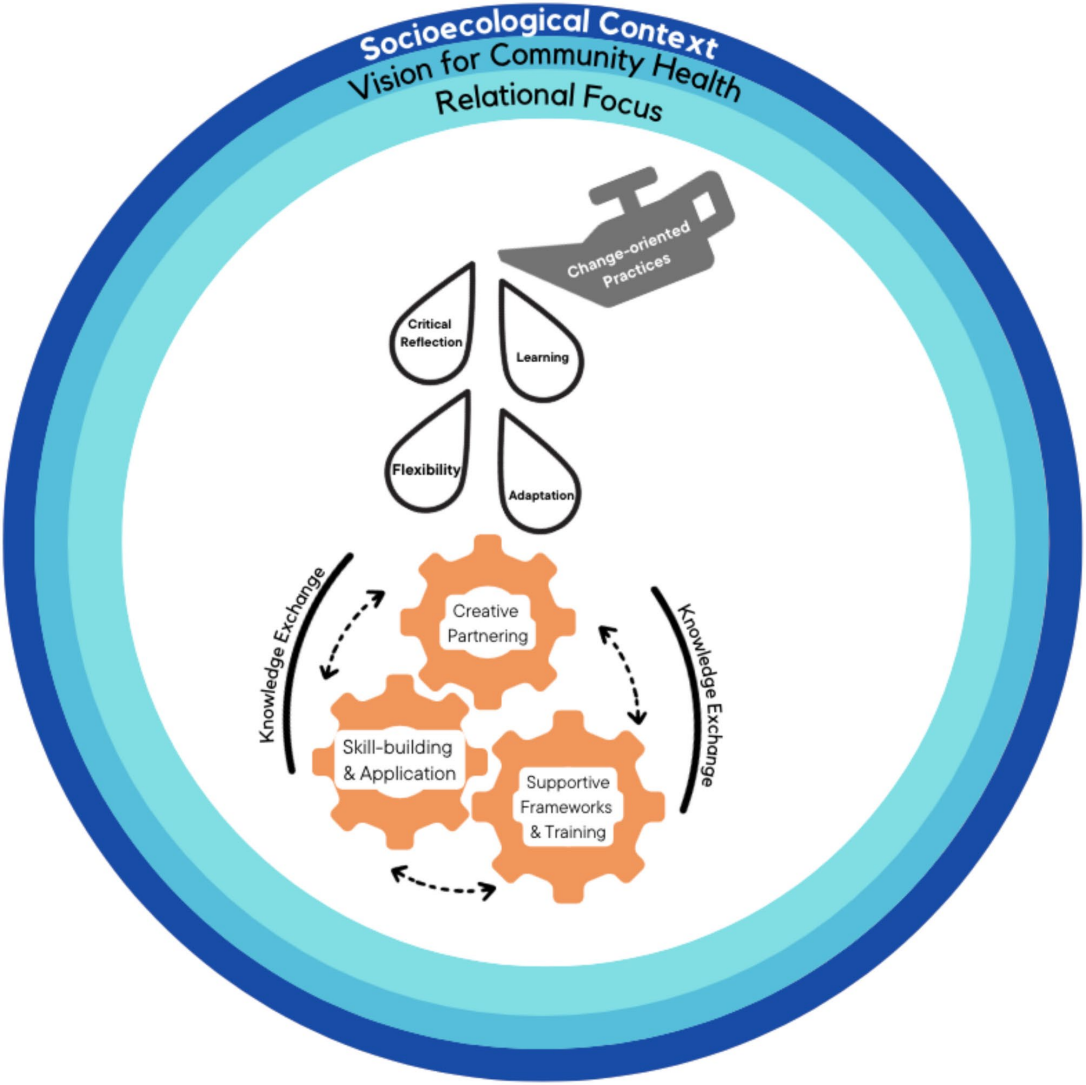
Knowledge exchange framework for trauma-informed practices, initiative best-practices and communicating community needs across agencies, partnerships and community members. *Note.* This knowledge exchange framework represents the perspective of agency leads over a three-year period. While the represented practices are partnership-based, which includes Community Ambassadors, the actual mechanisms (gears) of knowledge exchange include all community members

Figure 2 distills three broad contexts that surround knowledge exchange as identified by agency leads (i.e., socioecological context, their vision for the community they serve and a relational focus on achieving mutual goals) as well as the central components of a knowledge exchange framework related to trauma-informed practices, initiative best-practices, and communication about community needs that support cross-sector capacity-building. During the baseline interview, agency leads characterized the overall socioecological context within which the initiative occurred, including pre-initiative community trauma exposures and obstacles to well-being in their community. Barriers to well-being were discussed across all three interview waves, particularly with the onset of the pandemic prior to the mid-point interview. Details about their vision for a healthy community initially arose during the baseline interview and continued to unfold during the midpoint and final interviews. This vision included aspirational goals for their community, based on observed community needs and practical support for resiliency. Further, a relational focus on engagement, characterized by relationship- and trust-building, emerged for the initiative’s work. This theme was evident across all interview waves, organically arising during the baseline, and followed-up during the midpoint and final interviews [88]. See Table 1 for additional exemplary quotes.

Agency leads’ vision for healthy communities, together with a relational focus, set in motion important practices (oil can), and mechanisms (gears) which kept the initiative nimble while engaging their communities. Four essential practices (Table 2) kept key mechanisms in motion: (1) critical reflection on the INN2 initiative within their respective communities, (2) learning that was reflection-based and part of the initiative’s culture, (3) flexibility that became part of the partnerships’ mindset, and (4) adaptation - actively changing approaches based on what was gleaned from a reflection-based learning culture and flexible mindset. The underlying mechanisms (Table 3) that powered the initiative’s evolution in response to changing community needs and an emerging pandemic, included: (1) creative partnering to address resource and service gaps; (2) supportive frameworks and training opportunities for all stakeholders that facilitate multidirectional knowledge exchange; and (3) skill-building, including the direct application of those skills. Essential practices and key mechanisms underlying knowledge exchange were central themes across all three interview waves.

## Broad Contextual Factors Surrounding Knowledge Exchange

### Socioecological context

#### Traumatic experiences contributing to agency participation in INN2

Traumatic experiences and broad exposure to adversity was common across communities. Three themes consistently emerged across partnerships and strategies, reflecting key individual-level and systemic social determinants of health: (1) familial trauma, (2) community-based violence, and (3) systemic adversity.

First, familial traumas reflected child maltreatment experiences, adversity-driven maladaptive coping such as substance use, relational violence and intergenerational traumas, set against a backdrop of limited information about what constitutes a traumatic experience and the consequences of trauma, given the pervasiveness of these experiences.

*There’s child abuse [and] substance use. We find that with ACEs, we have a lot of community members that have Department of Children and Family Services involvement. [The parents] were foster youth themselves, grew up with a parent that was incarcerated or a parent that wasn’t around…. [T]here’s also neglect. A lot of our participants…don’t actually know what neglect is. [I]f we ask them “has anyone ever neglected you,” they’ll tell us “No.” But, if you start describing it, then they [say]“Oh yeah, that happened!”*

Second, community violence themes centered largely on gun violence and gang activity. Community violence was so common that it was perceived as a way of life. As one agency lead remarked: *A lot of the community members experience gun violence. [T]here’s gang activity. [W]e don’t see it as trauma*,* this is a way of life*,* it happens.* The pervasiveness of violence outside of the home was observed through intergenerational gang involvement, which impacted all regions in the initiative:

*[Our] community has a history of having some of the oldest gangs. It’s [where] all these movies have been made*,* so [community members] share that history. Not everyone’s a gang member obviously*,* but it is a viable option for our [youth]*,* something they are recruited heavily*,* since elementary [school]. That’s been consistent*,* so you have generational gangs.*

Third, systemic adversity was a key emergent theme across all partnerships. Poverty affected individuals directly, as well as indirectly, though reduced funding for schools and fewer community resources. Increased community stress also contributed to overall health disparities. All agency leads repeatedly noted important, cultural historic roots of trauma that contributed to the stress of their communities: community traumas, racism, structural racism, and historical trauma from the intergenerational transmission of violence, escaping genocide and/or long-term consequences of slavery. Over-policing was identified as common and long-standing facet of systemic adversity across all communities, with an extensive police presence leading to high arrest rates among the community’s youth which was further complicated by maladaptive coping strategies adopted by youth, such as substance use:

*[Our community was] heavily policed in the ‘90s and 2000s*,* and that’s kind of left it kind of where it is today. A lot of the seniors [in high school] that come through our programs have also been arrested*,* incarcerated*,* and/or they have a substance use problem. [T]hey all have their own [traumatic experiences] that led to these things [like arrest and substance use] occurring.*

Finally, nearly half of the agency leads described documentation status and the threat of deportation as increasingly comprising the trauma experienced by their community members:

*[N]ow with a lot of families that we serve…they’re just very fearful due to [their] documentation status. [A] lot arises [from] their concern for their children*,* or for themselves*,* or their parents.*

Despite these current and historic adversities, agency leads repeatedly noted the strength and resiliency of their community members:

*It takes a lot of resilience to be undocumented and still be able to thrive. To be able to sustain two jobs*,* and have your family*,* your kids going to school and doing really well*,* perhaps being in a Magnet or a STEM program. [T]here are those families here as well. [W]hen you have a lot of challenges*,* it takes a lot of resilience to swim against the current. We have a lot of families that show us that here day-in*,* day-out.*

#### Barriers to health and well-being

All agency leads reported barriers to health and well-being in their communities. The three most common themes were: (1) paucity of basic resources, (2) community distrust of government agencies, and (3) a need for an alternative approach to services. The most consistently reported barrier across all agency leads was the paucity of basic resources:

*[Our] perspective of the biggest challenges in the community’s health and wellness [are] the resources that are available. [T]here [are] tons of little liquor store markets*,* but…people are asking for a [real] market here…. [I]f you don’t have a car*,* you’re a mom with a few kids and you’re going to use the bus*,* you don’t have the opportunity to go really far to buy a bunch of groceries. [It’s] much easier if you’re tired to go across the street to [get fast food].*

Deficits were also noted for age-appropriate services, which are often overlooked unless explicit asset mapping is conducted because they represent populations with little economic power or voice within their communities: *[Our team] is always looking for resources to support youth*,* because there’s basically nothing in LA to help youth. There [are] so few resources compared to the number of adult services*,* and it’s so important to address youth houselessness*,* homelessness [and] trauma. [Helping] at-risk youth [is] preventing the next generation of chronically homeless people.*

Community members level of distrust was recognized by all agency leads as a barrier to health and well-being that needed to be overcome. Distrust was often caused by fluctuations in programming and resource availability, and negative experiences with social service agencies such as Child Welfare: *[We] did an ACEs screening pilot…and they found that a lot of the families [had] six or more ACEs. Those families [with] three to four were much more open to having services provided. But a lot of the ones that had six or more? There was a lot of distrust already built and they already felt like “I don’t want to engage with other providers” because they have [Child Welfare] involvement or other types of services coming into their home already and there was just a lack of trust when it comes to organizations.*

All agency leads recognized and underscored that community members were reluctant to reach out for help given a long history of public health programming eliminations:

*[W]e’re also mindful that communities have had so many programs go away. [The community starts to] question your intentions…. [W]e’ll have to build trust and rapport*,* and that’s going to…take time.*

The pernicious effects of negative community attitudes and stigmatization were also seen as creating distrust:

*[P]art of the community may not think people deserve quote-unquote “handouts” or help. A lot of times [when] people don’t have a personal experience with something*,* it’s harder for them to understand*,* and they’re like*,* “oh*,* I think like these people are dangerous” or “we’re just throwing money.*

Finally, several agency leads perceived the public’s desire to have their mental health needs met through alternative avenues as a potential barrier to community well-being. While community members’ interest in a different approach to address their mental health initially posed a challenge for the social service-oriented lead agencies, it also provided insight into alternative means of engaging the public and re-thinking mental health services from a broader perspective:

*There’s a need for nontraditional means of addressing mental health. [T]here’s money for people to get a Medical…and one-on-one therapy but youth don’t always want that. [S]ometimes they want to address their mental health when they don’t even know that they’re addressing their mental health*,* like healing arts program. It’s like the gateway to everything else*,* [something] that makes them feel more connected and [provides] a space to build those initial relationships.*

Taken together, the interconnectedness of trauma and adversity exposures, limited resources, public stigmatization and need for alternative approaches to mental health all reported by agency leads underscore the complexity, pervasiveness and scope of problems facing many contemporary communities. When viewed through this lens, it becomes clear how the same domains of resiliency (cultural, economic, environmental, political and social) may also converge to amplify disparities across the lifespan and intergenerationally. Greater consideration is warranted to understand and address how stigmatization and discrimination are likely to increase avoidance and shame in those experiencing adversity, which in turn increases risk for psychopathology, decreases the probability of seeking social support and potentially reduces resource utilization [102–106]. Finally, agency leads insight that large-scale social determinants of physical and mental health are unlikely to be ameliorated by direct mental health services alone is important and demands approaches that value knowledge and guidance from community members.

### Vision for community health

Agency leads were asked about a) their vision for their community’s health, b) the main thing that they were trying to achieve in their community and c) their aspirational legacies. Thematic saturation was quickly achieved. Agency lead’s answers did not reflect an exclusively strategy-specific vision, achievement, or legacy. Instead, their vision, achievement goals and aspirational legacies solidly centered around all levels of their community, including community members, government officials, local organizations, and businesses being trauma-informed, non-stigmatizing and supportive. Being trauma-informed included the hope that community members would be invested in their community’s overall health and proactive in problem solving: *[W]hat I really want to see is a community that’s invested in their wellness*,* and what I mean by that is to really*,* truly acknowledge how 1) trauma and different policies have impacted this community*,* and then 2) how do we reverse that?”*

De-stigmatization was perceived as a specific, clear step in reducing health disparities in the community:

*I would love to see the individuals who have this label of mental illness be able to get employed. I’d like to see them get a living wage just at the same rate as any other person in the population does. We are trying to change the way that the community views our neighbors experiencing homelessness. Not looking at them from the lens of fear or the stigma that surrounds homelessness*,* mental health and substance use but looking at [them as] from ‘they’re our neighbors’ and ‘how do we help our neighbors?*

Agency leads’ vision, goals and aspirational legacies included internal (within partnership) and external (with and among community members) objectives. First, they strived to not only equitably exchange knowledge about available resources but truly embody trauma-informed principles partnership-wide, highlighting that change is possible:

*[E]ducating the community at large about available services*,* not just through us*,* but through our collaborative partners as well. [C]oming at the issues with compassion vs. a ‘not in my neighborhood’ feeling. [O]ur overall hope for our legacy [is] through not only doing the community outreach and education portion of it*,* but really trying to walk the walk*,* not just talk the talk*,* and serving as an example of what a trauma-informed*,* culturally and linguistically sensitive program is able to accomplish in a fairly short amount of time.*

Second, they were striving for a community of members who are involved, autonomous and take ownership of the work itself so that these practices become organically rooted in the community:

*The main thing that we’re trying to achieve is…involvement from the actual community. [T]he voice of the community really being at the table. How can we build up community leaders and parent leaders that are already existing in the community so…they can continue doing this work when the contracts ends*,* [so] that it’s not [going to] just die with the contract. [I]t’s going to be something that’s implemented and the community takes ownership of it. [T]hat way they’re continuing to have events. They’re continuing to be able to have trainings around trauma-informed care.*

In these ways, the agency leads envisioned the partnerships taking active responsibility for not only exchanging knowledge with their community but incorporating the knowledge they were gaining within their partnerships while supporting the autonomy of their community members to act on this knowledge and take the lead in transforming their communities.

Notably, on the occasions when agency leads did incorporate their specific strategy into their overall vision for their community, the themes were similar and primarily encompassed the importance of: (1) strategy-specific community members understanding how trauma was transmitted (e.g., the intergenerational transmission of trauma in families with children under the age of five, who were at-risk for maltreatment) and (2) being autonomous and taking an active role in reducing strategy-specific community problems such as homelessness or providing job opportunities for individuals reintegrating into society after incarceration. See Table 1 for additional exemplary Vision quotes.

### Relational focus

Embracing a relational focus was a central theme that emerged organically at baseline and was consistent over all three interview waves as well as across agency leads. Creating environments that support healthy relationships requires openness and active listening. A critical first step in engagement was “meeting people where they are at” rather than expecting community members to seek out agency staff. As one agency lead noted:

*[I]t’s not just waiting for our community partners or even our community to come to us*,* it’s really going out and asking them what they might need*,* and actually really showing them that we’re invested in making sure that they’re healthy all around [and] making sure that they know about services and can link other people [in the community].*

A relational focus included providing engagement opportunities that address basic needs, regardless of whether these opportunities are a gateway to traditional mental health services. Providing basic services such as showers, laundry, food distribution, and housing addressed basic necessities and provided the opportunity to engage community members in needed mental health care: *[W]e’re trying to show that by meeting basic needs, we are also working towards their overall mental health and their physical well-being.* Healing historic distrust through social connections was central to the mission of a relational focus:

*[T]he people that we [work] with have learned to distrust “the system” for all of their life, especially aged-out foster youth. [T]hey’ve heard it so many times, it’s really hard to build that relationship. We have to go back to activities. You’ll see in our lineup there’s a lot more Table Talks, more Friendsgiving, a karaoke night. There’s spoken word. They don’t want to be lectured to. It’s more about building a relationship and a bond, which takes time.*

Trust-building provided the foundation for authentic relationships, engagement, and capacity-building within agencies, across partnerships and with communities [88]. Consistency was identified as a key aspect of being trustworthy: *Our trust is [based on] follow-through*,* doing what we say*,* following up and not disappearing.* Trust-building requires consistent communication and genuinely embodying other qualities of trustworthiness, such as transparency, reliability and competence, that similarly enhance safety. Authenticity was viewed as an essential component of trustworthiness, needed to build common goals, create a shared vision and address historic inequities, each of which improve engagement, support relationship- and trust-building, facilitate power sharing and enhance collective impact: *[It’s about] authenticity. How are you showing up? [W]hat does that mean in the way that you meet with people*,* engage and build relationships? [W]e’re really tackling these systems and these issues*,* so we can move collectively together.*

When relationship- and trust-building are successful, and engagement opportunities and resources are consistent with what the community values, the result is word-of-mouth, direct referrals among community members: *It’s really [about] building trust. [W]e have a really great team that is working on the individual-level trust-building*,* which then spreads*,* through word of mouth*,* which is probably our best referral source.* Overall, a relational focus provides a strong foundation for collective impact: effective implementation of an initiative’s vision, collaborative engagement and healthy knowledge exchange among trusted sources.

## Practices facilitating knowledge exchange

Across partnerships and over time, four key interrelated practices built upon each and kept the initiative’s work in motion. **Critical reflection** created the space for **learning** and allowed a learning culture to develop, while **flexibility**, specifically a flexible mindset when challenges and opportunities arose, set the stage for **adaptation** – permitting active adjustment to changing needs and circumstances and ultimately making transformations possible. Together these four practices created the conditions for facilitating the initiative’s work with intentionality – shared focus, attention and goals [107–109]. See Table 2 for additional exemplary quotes.

### Critical reflection

Critical reflection on the initiative’s work, including community strengths, goals, needs and barriers to wellness was central to effectively engaging communities, building internal and external capacities, and intentionally conducting the initiative’s work. A critical shift occurred internally as agencies reflected on the universality of trauma exposure and the need to apply their trauma-informed knowledge to their own staff and colleagues: *[A] lot of our work has been focused on how to work with clients from a trauma-informed care lens*,* but not with other staff members. [S]taff can also have histories of trauma that we have to be aware of. That’s one thing that we’ve done that’s really helped…increase knowledge and understanding of how we should be interacting with…the people we work with.* This insight helped to break down perceptual barriers between staff and community members through the recognition of shared stressful experiences. Notably, this insight was gleaned even during the baseline interviews, predating the pandemic and introduction of a more codified Community Ambassador Network.

Critical reflection also supports acknowledging the agency’s role within larger systems of care and creating a broader sense of “community”: [*R*]*eally being intentional on a system’s impact. [W]e’re focusing on “community” as an organization and individually [and] saying*,* “How are we being more intentional in the collaboration with the system?” [T]hat way it’s not us vs. them; or us then them; or them*,* then us.* This reflection extends working with intentionality to include a better understanding of potential unintended consequences. Specifically, seemingly positive ideas or frameworks can also have negative implications, such as placing the responsibility to make systemic changes on the very community members who are without power or resources: *We can get into a whole conversation about resiliency…and how a lot of the onus is being placed on the individual. Like you’re responsible for your own conditions and circumstances and if you just somehow work on your “resilience”*,* then you’re better off. [We need to] also look [at] these larger systems of oppression that make it harder for folks and are actually perpetuating these conditions in the first place.*

The pandemic solidified the idea that reflection should be built into the initiative, as “the work” is dynamic and requires ongoing monitoring of how partnerships meet evolving circumstances: *[The pandemic] was really*,* really challenging. But now today*,* we’ve now been rethinking [things] for ourselves like “How do we actually meet the work with where it is today?”*

### Learning

While reflection provided necessary insights, learning from those reflection-based insights was the next critical step in moving forward with the initiative’s work. The agency leads were grateful for the learning culture originally embedded within INN2 and embraced the “learn from your mistakes” model, extending it throughout their partnerships: *[T]hat’s been a blessing to have as part of this project*,* this room for error*,* this room for experimentation*,* this room for being innovative based on what we know from our training and our professionalism. [I]t’s allowed us to try things that we normally wouldn’t have.* Further, when learning dovetailed with a relational focus that included active listening, networks were more responsive to the needs of their communities: *We were given the opportunity to learn “what is it that’s going to be most impactful to community?” [A] lot of that learning came from listening to folks in the community asking for certain classes or hearing different stories and then saying “Wow*,* this is the theme here”. [I]t allowed us to learn and create programming that was responsive to [their] needs.*

Notably, the global pandemic exacerbated existing traumas and barriers to well-being (e.g., poverty, lack of resources) across communities, presenting an opportunity for partnerships to shift from their INN2 strategies and lean into their learning cultures to better understand, and meet, the changing needs of their community members. The experiential knowledge that resulted from navigating INN2’s role during the pandemic changed how agency leads thought about approaching their future work: *[N]ow*,* we’re also saying “[W]henever we do have to make a quick change or adapt to a situation*,* how can we actually have even more supports and teams behind [it]*,* allowing us to think and move and navigate that process?” [We’re] even thinking about “how do we…help prepare folks for that type of thing [in the future]?”*

### Flexibility

Flexibility – specifically embodying a flexible mindset - arose as a key practice against the backdrop of establishing a reflection-based learning culture. A flexible mindset was seen as an important part of being responsive, exchanging knowledge effectively, and learning from each other as well as from mistakes. This mindset increased a sense of responsibility towards what was shared during conversations with the community. Flexibility allowed the partnerships to be more creative in their approaches to addressing challenges, while being more community-centered and community-driven: *[T]hat sense of community was really helpful in having resiliency and not being afraid of change…. “[I]f it gets too comfortable*,* then we’re not growing.” So any time there was a shift*,* it was like: “This will allow for us to be stronger*,* for us to know better how to serve people*,* to know that it won’t all fall apart if a certain thing changes or doesn’t come through in the way that we expected. [W]e have the ability to regroup.”*

### Adaptation

Action-oriented adaptation allowed partnerships to build on information generated from their reflection-based learning culture, their flexible mindset and knowledge exchanged with community members, transforming how they engaged people and responded to the needs of their communities. The pandemic became a catalyst for adaptation that was supported by practices established early in partnership development: *When the pandemic happened*,* because we had this opportunity [to be a learning culture]*,* had this flexibility and were used to using it*,* it really allowed us to be extremely and accurately responsive to the needs of folks during the pandemic*,* beyond a food voucher*,* really programming content and approaches that would resonate with folks. [T]he pre-pandemic work*,* the experimentation*,* the learning that took place*,* and listening to the community*,* [all] set us up for success during the pandemic because we had established all that. We were comfortable with learning*,* not only from each other but from our participants [and] community leaders. [W]e created content programming. [A] program that was responsive during the pandemic resonated by evidence of having*,* in one of our classes*,* 60 to 80 people weekly from the community.*

## Knowledge exchange mechanisms

Agency leads consistently mentioned three specific mechanisms that powered the initiative’s work. First, in order to meet community needs and aspirations, the lead agencies creatively partnered with organizations embedded in, and respected by, their communities. This was particularly important given community members’ strong interest in alternative approaches to address their well-being and mental health needs. Second, agency leads embraced evidence-based frameworks such as trauma-informed practices, extending them to their own agencies and partnerships, while also incorporating other relevant trainings, frameworks and approaches that placed knowledge in the hands of stakeholders. Third, agency leads understood that the skills learned through these knowledge exchange opportunities had to be put into practice to facilitate meaningful impact. Table 3 provides additional exemplary quotes.

### Creative partnering

To build collaborative partnerships, agencies recognized the need to first establish their own credibility and relevance with community groups and organizations. Agency leads sought out partnerships with a wide range of organizations that had grassroot credibility to better reach community members and get the word out about resources:

*We’re part of the Homeless Coalition [and other] neighborhood coalitions. Other groups that we would love to have a connection with are multi-cultural organizations or specific cultural organization like the United Cambodian Coalition because they are such a big part of [our community] and we want to reach out to those populations as well.*

*[W]e started reaching out to some inter-faith groups and we want to build our network of inter-faith groups because some community members rely a lot on their religious leaders, their faith leaders. [I]f we’re connected with [them, we can let them know] “Hey, we have this safe space for Transition Age Youth” or “We have these employment resources.”*

A wide range of partners were incorporated into the initiative’s networks to provide functions ranging from referrals and linkages to direct services. This creative partnering did, however, pose challenges to DMH’s accountability metrics (e.g., confidentiality, financial documentation, memorandums of agreement): *Every organization is fantastic*,* but I think the commitment level to see this through [is essential]*,* the commitment to have*,* hire and train staff that would be able to be dedicated to this project. [I]t’s no easy feat working with the Department of Mental Health to pass their expectations and requirements.*

Despite these challenges, the pandemic highlighted the importance of partnerships and reduced “silo” approaches characteristic of many agencies:

*We were lone wolves before [the pandemic]. [H]aving something that allowed us to have subcontractors really opened our mind [to how we approach partnerships]. [N]ow*,* it’s a passion. [W]e love fostering these relationships and being able to mentor smaller nonprofits. [W]e get to partner*,* and [not be] afraid of the knowledge that other people have: ‘I’m glad that you know more about this*,* and that you can apply that skillset and knowledge to our agency.”*

The pandemic also leveled the playing field in many ways, permitting agencies to embrace their specific skill set while partnering with a wider range of organizations with skill sets outside of those usually connected to mental health services in order to meet emergent needs in their communities:

*[I]t’s broken-down barriers*,* knowing that it’s okay to not have all the skill sets ‘in house’. We’re not going to be able to do everything*,* but it’s great to know that there are people in the community who can. We aren’t going to be the ones to bridge that tech divide [need for Wi-Fi during COVID-19]*,* but we know people* *now* *who could.*

A flexible and adaptive approach to partnering allowed the initiative to reach more community members than is typical for traditional mental health service organizations. The agency leads’ view of not only viable, but essential, partners expanded. An important aspect of creative partnering, coupled with a reflection-based learning culture and flexible mindset, was the ability to pivot (adapt) during the pandemic, resulting in what agency leads called INN2’s universal “*COVID strategy*.”

### Supportive frameworks and training for capacity-building

Establishing supportive frameworks and providing relevant, ongoing training laid the foundation for the initiative and leveled the playing field for staff with a range of life and educational experiences. The primary focus of INN2 was infusing trauma-informed practices and knowledge directly into the community: *[T]rauma-informed care [gives] you that lens that whoever you provide support to*,* they might have gone through a difficult time*,* trauma or abuse in their life. [T]hat’s why it’s important to be kind and to be respectful…to not take things personal because it’s not necessarily a reflection of who you are*,* it’s reflecting their experience or something that may be triggering them. [F]or new staff [we’re] making sure they receive those kinds of trainings…so [we’re] investing in the professional and personal development of our staff.*

Other supportive trainings and frameworks rounded out the skills and knowledge base of community members and partnerships. These included Mental Health First Aid [110] which provides knowledge about recognizing and responding to mental health concerns directly to community members; the Community Resiliency Model [36] which provides skills for individuals to support their own resiliency as well as the resiliency of others within their social network; reflective supervision which focuses on relationships, rather than service-oriented compliance; and whole person approaches, which supply a framework for working with individuals experiencing complex problems: *INN2 has given us training that we would've not had to access otherwise. [O]ur para-professionals were able to go through mental health first aid. Everybody in the world should be able to take, and be certified in, mental health first aid. [I]f we could all recognize when other people are struggling, before it’s a crisis, that would change things for our community*. Together, these training opportunities provided a rich set of shared skills, evidence-based knowledge to Community Ambassadors, and a common language about resiliency, mental health and trauma across community stakeholders.

### Skill-building and application

Supportive frameworks and training opportunities provided the building blocks for applying specific skills that facilitated connections within the partnership and with community members. Skill development also builds capacities, creates professional opportunities, and facilitates autonomy by involving community members directly in the work. Sharing resiliency stories proved to be especially impactful for community members:

*One tool from the Community Resiliency Model is the power of their resiliency story. Not only do all of our Community Ambassador Networks and team members that have taken the Community Resiliency Model write out their Community Resiliency Model but when they’re facilitating*,* they share their Community Resiliency Model [and] their resiliency score. [E]very time we work on [a community] presentation*,* we do a run through*,* and one of the important factors that we always mention is: just speak your stories. Speak on how you use Community Resiliency Model. Speak [about resiliency] at a community-level that they will understand because that’s what we want to do - normalize these conversations.*

Population-specific skills helped partnerships reach individuals who were disenfranchised and provide them with greater support, even in the face of political or cultural climates that reduce receptivity: *Our therapists have taken more classes in working with transgender [individuals] and dealing with transitioning. We had never*,* as an agency or a community*,* done that before INN2. I’m really proud of that work. It’s been slow going for us*,* because we are in a conservative community*,* but without INN2 I don’t know if we would’ve gotten there. And slowly*,* slowly*,* but each month*,* I notice that segment of our population and our referrals increasing.*

Overall, these skills have many real-world applications and, when developed within a safe space, permit time to reflect on how people have successfully coped in the past:

*INN2*,* during the pandemic*,* created spaces where people log in [and say] “Como estas? How did it go? [T]hey’re talking about things in the context of living under a pandemic. It opens [people] up. We talk about the importance of talking about things*,* including their support system. [B]ecause we’re having these conversations it’s nudged the door open for them to talk about other traumas in their life and the skills they’re using now to endure THIS trauma.*

Notably, learning was extended to reflections about how partnership and community members could adapt to future challenges:

*[W]e let people know in a very gentle and trauma-informed way*,* that the reality is that adversity will rear its head upon us as a community somewhere down the line in the future. Might be an earthquake here in Los Angeles. It might be social unrest. [H]ow do we take what we learned now*,* through the Innovations Project [about] dealing with trauma as a community*,* and hold it within us so that when we go to that next adversity chapter in our journey*,* in our life*,* we’re able to implement what we learned here?*

## Putting it all together

A practical, and strengths-based, example of putting this knowledge exchange framework into action is the incorporation of community members directly into the initiative’s work. The inclusion of staff with lived experience, including direct experience of individual-level traumas, community-level adversity and past system involvement (e.g., Child Welfare System) as well as typically being residents of the very communities that they served during INN2, was consistent with the agency leads’ vision of an informed, resourced, non-stigmatizing community: *[O]ur Community Ambassador Network interns are phenomenal. They’ve been in systems. They’ve been in the resources that we are providing and are able to navigate them a lot more. They have the resources [to work with the community] under their toolbelt.* Staff with lived experience also exemplified the relational focus of “meeting people where they are at” and building trust *[I]t’s peer-to-peer*,* people from within the community that are actually doing this work. The teams that are serving the community are reflective of the community*,* which definitely helps with trust and safety.*

Staff with lived experience further underscored how multi-directional knowledge exchange can value both content expertise, such as capacity-building, trauma-informed practices and other professional skills, and lived experience - context expertise - about community needs and community strengths:

[The Community Ambassador Networks] are still very much dealing with a lot of [COVID-related] stuff that we’re all navigating. [T]heir voice at the table has been critical to help us better understand our participants, better understand the dynamics, better understand if there’s a community event or something happened like an incident, they can [tell us] how it’s really being discussed in the community.

The direct input of staff with lived experience transformed the initiative’s work and organically resulted in power sharing: *[W]orking with our interns has amplified the work that we’re doing because we’re learning from them as well. Even though this is [their] mentorship*,* it’s also a mentorship for us because they live in South LA. They have that background*,* that experience and can help us navigate who to outreach to*,* who to bring these resources to*,* and what resources are missing. [T]hat’s why community involvement is very important. [The Community Ambassador Network is] part of the decision making.*

Incorporating community members directly in the initiative’s work reflected flexible and adaptive practices as well as creative partnering, with shared training opportunities and skill-building for staff with lived experience. This approach also provided opportunities for the broader community to engage directly with people who had firsthand knowledge about their experiences and struggles, as well as trauma-informed practices and community-based resources.

## Discussion

The present longitudinal qualitative study provides insight into the inner workings of a real-world, collective impact initiative that builds capacities through a trauma-informed lens. While recognizing the resiliency of community members in the areas they served, agency leads also identified traumatic experiences and perceived obstacles to community health, alongside their resulting vision for community health while reflecting on the practices and mechanisms that facilitated trauma-informed knowledge exchange to support community resiliency. Knowledge exchange included extensive trauma-informed trainings partnership-wide (Supplement Table 1), community engagement opportunities and trainings (Supplement Fig. 1), word-of-mouth referrals and resource sharing among community members themselves, and relevant referrals and linkages for community members from the partnerships (see also Gilmer et al. 2021) [87].

Among agency leads, the most important examples of multi-directional knowledge exchange was incorporating community members directly into the initiative’s work through the Community Ambassador Network. These individuals contributed context knowledge of their communities directly to the partners, while their lived experience as residents within these communities; and/or prior system involvement, mental illness, adversity exposure or experience using community-based resources enhanced credibility within their community as they provided social support and relayed information about the consequences of trauma and how to identify different forms of help [86]. Through the initiative, the community ambassadors also gained professional skills and training from the lead agencies as well as a range of partners and external mental health experts. Notably, DMH observed the resiliency of the community members in Los Angeles early in the initiative and amplified this strength by creating the Community Ambassador Network. This approach proved particularly invaluable during the pandemic, as the ambassadors constituted a critical social infrastructure for their communities; they were well posed and trained for outreach and engagement, including listening to their fellow community members and working with partners to directly address community needs in real-time.

Despite the community members’ inherent resiliency, traumatic experiences occurred at familial, community-wide, and systemic levels, with frequent overlap. Familial trauma was relational and intergenerational in nature, intersecting with systemic adversities such as stressful immigration experiences. Community violence, particularly related to gangs, was similarly characterized by intergenerational transmission and co-occurred with systemic traumas such as incarceration and discrimination among disenfranchised citizens across all communities. The types of trauma reported, including broad systemic adversities such as poverty, were consistent with data from urban and rural communities in the US and other countries, underscoring the pervasiveness of social determinants of health and relevance of culturally attuned trauma-informed practices for serving the needs of many communities [1, 111–113]. 

While agency leads noted the remarkable resilience of their community members, they also reported specific consequences of trauma exposure that may further impact health-risk, including trauma-driven maladaptive coping strategies such as substance abuse, increased distrust as trauma exposures accumulated, and a sense of resignation that trauma is “just daily life” among community members. Adversity-driven maladaptive coping strategies are also social determinants of health that directly (e.g., substance use) and indirectly (e.g., through associations with arrest and incarceration) amplify health disparities. These strategies are associated with more severe trauma responses and increased risk for new trauma exposures, are consistent with posttraumatic risk-seeking common among individuals with developmental trauma exposure and overlap with Posttraumatic Stress Disorder’s ‘reckless and self-destructive behaviors’ diagnostic criterion [114–119]. 

Distrust of individuals and systems is also associated with developmental traumas such as childhood maltreatment, and systemic adversities such as marginalization, discrimination and disenfranchisement [120–122]. In turn, distrust reduces help-seeking, creating additional barriers to resource access, utilization and follow-through while increasing negative health consequences and disparities [123–126]. Finally, while normalizing conversations about the health consequences of adversity and trauma appeared to raise awareness, diminish stigma and reduce isolation, the duality of “normalization” was also observed. Specifically, the perception that trauma was simply a way of life and normal, was particularly apparent for experiences such as childhood neglect. This has clear implications for family health, the intergenerational transmission of trauma and lowered resource access: when normalization means a lack of awareness, that normalization perpetuates adversity across generations and impedes help-seeking that may offset the negative impact of these experiences.

Agency leads also identified non-trauma barriers to community health and well-being, such as a lack of basic resources. Job opportunities, non-predatory financial institutions, grocery stores and safe public spaces were critical needs that community members conveyed to the partnerships. Stigmatizing perceptions held by the larger community about members needing public services were also appreciated as a barrier to overall community health. Notably, stigma, and stereotypes were not restricted to mental illness or the effects of trauma, but included broader considerations such as how public funds were used and generalized negative perceptions about public fund recipients. Agency leads initially perceived public interest in alternative approaches to mental health that prioritized social connectedness, rather than traditional mental health services, as potential barriers to community well-being. However, over time they came to view this as an increasingly important opportunity for shared knowledge, power and experiences, highlighting the importance of muti-directional communication and adapting to community needs. The pandemic also shed light on the central importance of technological services such Wi-Fi and computer skills to support basic needs, healthcare and social connections for all community members, as well as education access particularly for youth and young adults. These layered and interconnected services and needs were crucial for mental health and well-being.

Taken together, the micro (familial), mesa (community-wide) and macro (systemic, political) trauma and barriers to health presented substantial obstacles to overcome. The obstacles described by agency leads aligned with characterizations of social issues as intractable “wicked problems”: [127, 128] complex, costly, interconnected large-scale dilemmas that foster disagreement about both the origins of, and solutions to, these problems and highlight conflicting values. Given the pervasiveness and intractability of trauma and adversity, alongside community members’ perceptions about the lack of resources, it is unsurprising that agency leads’ vision for their communities were largely centered around connecting community members to existing resources and identifying additional resources beyond traditional mental health services, through creative partnerships. Interestingly, agency leads did not report that their goals for their community were specifically centered around reducing traumatic experiences. Rather, they emphasized the importance of getting information – particularly about the impact of trauma alongside sharing skills and resources to offset the negative consequences of adversity - into the hands of community members, while supporting community resilience and empowering community members.

This is a practical approach to envisioning healthy communities, given that systemic issues such as widespread poverty and institutionalized discrimination require effective policy changes, public and government buy-in and adequate funding, all of which fall largely outside of the control of social service-oriented agencies and impacted community members. This idea was further underscored by agency leads’ acknowledgement that even efforts at promoting “resilience” may place the onus of change on disenfranchised individuals. For these reasons, the initiative’s empowerment efforts focused on sharing knowledge with partners, community groups and other agencies about how trauma impacts physical and mental health, and strategies to destigmatize the affective and behavioral fallout related to trauma and broader adversities, while enhancing help-seeking at the level of their community members.

To achieve their vision of a healthy community, agency leads embraced a relational focus on engagement. This included meeting people “where they were at” emotionally and physically within their communities as well embracing broader engagement opportunities that support mental health through social cohesion. Community-based activities, such as knitting circles, opened the door to deeper conversations about what community members need and value most, creating opportunities to increase a sense of belonging and breaking down barriers between service providers and community members. Foundational to embracing a relational focus was the prioritization of relationship and trust-building, with a deep understanding that authenticity was central to engaging both community and partnership members. These organically arising insights align well with data indicating that community engagement, characterized by prioritizing collaborative relationships built on trust and shared decision-making, may be the most effective, or powerful means to promote community health and social change [129]. 

Agency leads also identified four interconnected practices that allowed them to be nimble: critical reflection, a culture immersed in reflection-based learning, flexibility, and adaptation. The importance of these practices was tested during the pandemic when all partnerships shifted to a “COVID strategy” characterized by providing for the basic needs of their community members. Consistent with fundamental practices for professional development that support creative problem solving and “learning organizations” that interpret experiences and adapt as part of their culture [130, 131], critical reflection allowed the initiative’s work to be done with greater intentionality, a luxury not always permitted within initiatives where funding restrictions are emphasized rather than innovation, nor characteristic of social service agencies operating on limited budgets and often understaffed [132–135]. Critical reflection has previously been identified as an important capacity in support of public health that challenges assumptions, analyzes underlying values, and integrates concepts of social justice in the service of equity-based change [136], aligning well with the perspectives reflected by INN2 agency leads. A reflection-based learning culture included learning from the community, staff with lived experiences and other partnerships, including quarterly learning sessions for cross-pollination of best practices. The ability to “fail” and learn from their mistakes provided further opportunities for problem-solving and testing novel approaches.

A flexible mindset included an openness to hearing what the community and partnership members needed, even when what community members needed most, fell outside of traditional mental health services. This mindset set the stage for action-oriented adaptations to address changing community needs, including the rapidly escalating public health circumstances during the COVID-19 pandemic. Together these four practices reflected the importance of both internal, contemplative processes such as mindset (reflection, flexibility) and more action-oriented processes (learning, adapting), creating the conditions to facilitate the initiative’s work with intentionality while mitigating the friction caused by obstacles and unanticipated circumstances.

These practices kept in motion mechanisms that democratize knowledge exchange through shared training and frameworks that enhance engagement and promote transformational environments. These opportunities support skill development across stakeholders who become fortified with trauma-informed knowledge and practices that enhance resiliency and reduce stigma. An evolving and creative partnership, adapting to ever-changing community needs, was central to the vision of placing knowledge and resources directly in the hands of the community.

### Limitations

The present framework underscores practices and mechanisms that support knowledge exchange in collective impact, urban capacity-building initiative and provides insight into practical steps for future consideration and refinement from other projects, as well as community members and scientists representing different cultural perspectives. While the framework is supported by data representing different strategies tailored to address specific trauma-related needs for a range of diverse subcommunities, it would be strengthened by perspectives from rural regions, other countries, and a broader range of stakeholders (e.g., government officials, funding agencies), especially community members, as the present data are limited to leadership perspectives. A strength of this work is that it represents a real-world initiative, not driven by researchers or academic-community partnerships. This means, however, that we focused on understanding how agency leads thought about, and hoped to address the trauma-related challenges that their communities were facing. This may underplay the resiliency of their communities. We further acknowledge that we did not explicitly ask for each agency lead’s definition of the terminology that they used (e.g., resiliency, well-being), or the explicit level of their terminology (e.g., individual-level or community-level resiliency), both of which might be of interest to various readers. As such, we cannot adequately address these potentially contested terms in the literature based on the presented data. Finally, mixed-method data from other community-based initiatives that approach community health and capacities from an equity-based perspective would strengthen this framework.

## Conclusions

The socioecological context of familial, community-based and systemic traumas, combined with barriers to well-being, informed agency leads’ vision for community health and approaches to capacity-building. Agency leads prioritized a relational focus for community engagement and outlined an initiative-wide framework for knowledge exchange across partnerships and over time. Fundamental practices of critical reflection, learning, flexibility, and adaptability emerged alongside practical mechanisms (i.e., creative partnering, supportive frameworks and trainings for capacity-building, skill development and application) facilitating multi-directional knowledge exchange across stakeholders for healthier, better-informed communities with the potential for greater resiliency in the inevitable face of future adversities.

While the specific trainings, frameworks and partners needed may vary across initiatives addressing unique issues, or implementing specific programs in distinct regions or at different timepoints, the importance of retaining – and continuing to exchange - knowledge from training opportunities, integrated frameworks, and skill development should be underscored as institutional and partnership memory are key to sustaining advances made during any initiative. Historical knowledge, flexibly and adaptively applied, is critical to serving communities, as needs are always evolving. Especially in the realm of public health work where staff turnover, changes in partnering organizations, and emergent circumstances (e.g., funding loss, health crisis) are common, retaining and exchanging knowledge in a way that can be flexibly applied allows partnerships and initiatives to remain nimble – not only adapting but transforming the work and their communities as circumstances inevitably change. The framework that emerged, and the example of incorporating community members with lived experience directly into the work of a public health initiative, underscored the value of both content and context expertise, provided a voice for community members’ input into the initiative’s work and increased opportunities for power-sharing. Taken together, these findings align with factors previously found to impact healthy, change-oriented socioecological trajectories: resilience, adaptability, and transformability [137]. 

**Table 1:** Socioecological context related to community needs, agency vision for community health, and relational focus

**Socioecological Context**	
Traumatic Experiences	Obviously, child abuse. We’ve had a lot of high-profile child deaths that dealt with physical abuse.
	When you’re looking at African American, Cambodian & Latino, a lot of it is historical: racism, genocide & slavery…I think there’s the community traumas, the racism, the structural racism, the historical trauma that’s both from a violence or coming out of genocide or coming out of slavery and those traumas.
	[M]any of our staff are from the community we serve and have intergenerational [trauma]. [G]ardening is very big for our Cambodian [population], very healing for them, because many [grew up on] farms. [M]ost of the highly educated people in Cambodia were killed. So many of the Cambodians that [are] here were never educated, can’t read or write and gardening is their healing. I was telling one of my [Cambodian] staff that I visited [a community member] and got to see her garden and talk to her. [I]t was really special. [Our staff member] got tears in her eyes because that’s what her parents did. She [said] “*I used to be really embarrassed by it. [I]t was really sweet [but] I was really embarrassed by it. [N]ow I realize that’s what connected them to where they came from. [T]hat’s what made them heal in many ways. Now*,* I really respect what they went through*.”
Barriers to Health & Well-being	[P]overty out here is traumatic. We have parents with their kids who are panhandling in parking lots. [That’s] traumatic for the kids [and] traumatic for other community members.
	[T]here are more liquor stores & fewer parks in our community. Why is that? [I]t’s peeling back the layers & having conversations about racism. [N]ow with the tax against undocumented communities, it’s also talking about that. You can’t talk about trauma without [that].
	It’s really important…to hear what these communities are saying: “*I’d like to get a job*”, “*I’d like to feel safer*.” “*I’d like the park to be nicer for my kids*.” “*We don’t have a bank in our neighborhood*.” God, that one blew me away. Or a nice supermarket. Community center hubs in parks with…people that…give behavioral health information, along with how to get a reduced fare driver’s license or resource[s].
**Vision for Community Health**	
All community members are trauma-informed (e.g., public, government officials, businesses)	[Our vision] is more with our partnership [than] the community itself, but we haven’t really had that with our elected officials. Ultimately, that is the goal: to have those conversations, engage elected officials and [them] really assessing and reflecting on how they respond. What could they be doing to prevent, instead of create, more adversity? [L]ooking at how they’ve responded to COVID, even their own policies, how community members are left out of those conversations and the decision-making process, and how that’s harmful. Ultimately, that’s the aim, to start having conversations about how that’s definitely not trauma-informed. What could be changed? What could be done to form a partnership between community members and elected officials?
Community member autonomy	One piece that we lean forward into more now [as our vision] is enhancing leadership development & skills. [O]ffering opportunities for further applying those leadership skills [and] providing community members with opportunities to be actively involved in their neighborhood councils. So, how can they participate outside of INN2? [T]hat’s my vision, what I’m hoping is the next level. As we enter into the new year with the neighborhood leadership, neighborhood councils, what those represent, how much power they have in communities and the decision making that they have, and how representatives from the community can [work] from those positions that are more local, civic engagement. How are we providing other opportunities for community members to become involved in further applying their leadership [skills] and being part of the decision making that affects their own communities locally
Community ownership of the work	I [am] looking forward to…entering this new phase with the leadership groups. [T]hey’ve been expressing, and we’ve observed, there’s a need now to move more into advocacy. How they influence and impact policy. [S]ome of them have, on their own, been involved. I think we’re finally at this point where we have learned what’s been working, what hasn’t, and where we’ve had the greatest impact.
**Relational Focus**	
*Relationship-building & Engagement*: Safe spaces and Help-seeking support	[C]reating spaces where it’s okay to ask for help, or hearing other people say, “*Oh*,* I went there and they helped me with this.*” Our strategies create spaces for not only learning from each other, but learning within ourselves, that it’s okay to ask for help. [T]he strategies have really created spaces and opportunities where it’s safe to ask for help, and that it’s not frowned upon. The discussion of help and support is always ongoing, so it’s become normalized. In communities where asking for help is stigmatized or seen as “*you don’t have it together*” or “*you shouldn’t be doing that*,* because that’s only for people who are crazy*”, creating spaces where it’s okay to ask for help is hugely impactful. [T]he goal is that hopefully families internalize that ability and pass it on to their kids.
*Relationship-building & Engagement*: Safe spaces *Trust-building*: Diversity and collaboration	We’re a very diverse group. I think every meeting is a learning experience. I think every meeting that we have, every collaboration is definitely a step towards trust-building. [B]eing diverse is one of the best things for our work in South LA because we all bring different input, different resources to the table, but making sure also that with safety, we’re providing those brave spaces in our meetings and making sure that…there’s no wrong or right answers. Going into these spaces with “*we want to hear what the community has to say. We want to hear what our partners have to say*,” and “*How can we better collaborate? How can we better support each other?”* [H]aving that brave space can definitely be one of the elements that builds our trust with our partners.
*Trust-building*: Transparency	[W]e don’t want to be in the position where we have to take back something we said. The same thing [is happening] now, in terms of the Community Ambassador Network program. We hear [from DMH] that the Community Ambassador Network program might roll over, but we are framing it to the Community Ambassador Network individuals that are on board as “*this is going to end on the 30th and we don’t know yet what’s going to happen. If something is different*,* we’ll let you know*,* but what we’ve been told so far is [this…]”.* That’s the most trauma-informed way of letting people know. We’re not giving you hope that you have a job in January [or] hope for anything that I can’t fully fulfill, because it’s in DMH’s hands…. Building trust by being honest.
*Trust-building*: Transparency, accountability, inclusive culture, shared vision and collective decision-making	[W]e’re being more upfront about where we’re at and what we’re doing. Everyone now has to be accountable. You’re going to get feedback from our peers because we’re all working collectively to get this work done. [Staff are] like “*Oh*,* we can [give leadership] feedback?”* and I say “*Yes*,* and we have to implement it. Here’s this committee who makes recommendations on how we are or are not achieving our goals and you can be a part of that committee.”* [I]t’s all these different pieces we’re doing to really help build further trust and bring young people into the work that we’re doing, to have staff with lived experience be part of the decision making. Providing innovative ways to get folks aligned and a part of sharing the work and vision that we’re trying to have here collectively.

The use of italics signals a quote embedded within a quote

**Table 2:** Practices that fuel for knowledge exchange and engagement efforts

Critical Reflection	[[M]aybe I can share more about partner-to-partner reflection and learning. We have dedicated conversations around [how] everyone was transitioning during [the pandemic] and [trying to learn] what the best practices were that each organization had found. So, really sitting back and giving people time to actually reflect on what they’ve been doing, because changes came so quickly that there wasn’t a chance for a lot of people to actually realize all of the great work and pivots that were being made. As far as reflections, it’s having more space, and more communication, with our INN2 partners about what they’ve been feeling and doing over the past year or so.
Learning	[A]s a result of what we were hearing, we’ve brought on [a new partner] and they’ve been really doing work around housing and tenants’ rights. [T]hey’ve been providing workshops [for] community members - particularly with so many evictions happening, these relief programs ending and the moratorium on evictions expiring. [T]hey’ve been providing these ongoing workshops once a month to all the participants and the neighborhood leadership groups and so, that learning culture has also allowed us to see what are the real issues that are happening and how can we be intentional in bringing on partners that we wouldn’t have thought about bringing on before. [P]art of the trauma that’s created is not having a house. [H]ow do we address that by all supporting the folks that are working and they have a specific campaign, which is like – I think it’s called Housing LA or I can’t remember the exact term, but their whole campaign is around ensuring that members who are at risk of being evicted know their rights and know how to prevent that, or at least get support and assistance on how to challenge that, or know their legal rights and have that legal assistance. So, part of that learning culture has allowed us to hear and listen to the real issues that are happening, and then find partners that can support us in addressing those [needs] directly with community members. [T]hat was part of the needs assessment during COVID: [which] participants [had] urgent needs? [When] we did needs assessments, housing became one of those needs that community members shared pretty consistently across the board
Flexibility	[T]he big lessons are remembering that you can’t be everything to everybody, remembering to be flexible. [M]ost folks are, but to be flexible in the way that life is going to throw you. [B]ig life is going to throw you huge things and you don’t have to have all the answers, but you have a group of people or a team around you that you can count on to help you come up with those answers
Adaptation	We just did so much pivoting that now it’s kind of second nature. [I]t was cool to be able to develop a skill like that. [W]e have the control to choose how we react to a situation, and we can either panic and sit back and let life happen, or we can say “*how can we attack this from a different perspective? How can we pivot? How can we better serve?*” [W]e did a lot of that. We weren’t doing grocery delivery [as part of our strategy before COVID] & we just added it in, because that’s what the community needed. So now it feels like you’re able to really adapt in that way.

The use of italics signals a quote embedded within a quote

**Table 3:** Mechanisms underlying knowledge exchange and engagement efforts

Creative Partnering	We [built] partnerships with small businesses [and] restaurants and provided funding to the restaurants. Then [we got] vouchers or gift cards through those restaurants and [were] able to give those gift cards to community members in need. We did that with local restaurants that represent the different communities, the different ethnic backgrounds of the community members that we service. And same thing with grocery stores. Instead of maybe going with our Ralph’s, we decided…”*Hey*,* here’s the local Cambodian grocery store that everybody goes to. Let’s get vouchers from them and let’s provide funding so we can help them during this difficult time*” or “*Okay*,* who are the small grocery stores that service the Latino community? Or the ones that are tailored more for the black community?”* And the same thing with the grocery stores that we chose that tailored their products to the Asian-American and Cambodian community. So [that’s] providing support to local restaurants. Sometimes it becomes more difficult to do that. Also when we have events, choosing to go with a vendor that is a local. If you go with an established vendor, they’ll bring their truck, deliver everything and that’s fine. But, what about going to a smaller restaurant that maybe can bring some of their staff and be the ones catering this event in the community? So the focus is on providing support to small businesses. [That] has been a lens that we’ve had from the beginning.
	We have our Community Ambassador Network program, which do the outreach, our action teams & we also have a [Spanish] group to voice that Latino voice – predominately women. So, the Latina women bring more of that leadership role in the community. It’s always keeping in mind how we empower the community. How do we uplift the community? How do we provide space for them? A lot of the women…have these tools. They have these resources, but they don’t have the space to bring them.
Supportive Frameworks & Trainings	[W]e got six staff trained and then they’re going to train the rest of our staff. [I]n general, the ‘train the trainer’ model is comfortable here. We’ve been doing that for years with evidence-based practices. [W]e love anything with the train the trainer model for sustainability
	Oh! The Community Resilience Model, the capacity build trainings, the reflective supervision. Our team is now going to take on the training for emotional CPR. These are all great tools that they’re learning when they’re out in the community, but most importantly for themselves. I want to keep that moving forward – making sure that we’re prioritizing ourselves whether we’re out in the field, whether we’re at home, whether we’re facilitating. [T]hat’s one huge factor that INN2 has definitely integrated into the work that we’re doing.
Skill Building & Application	[I]f the Community Ambassador Networks are able to carry that [education] into their day-to-day life - or even be trainers - then they can be leaders. [T]hen they get jobs in different agencies, they have these skill sets, and we’ve really become a way to build their resume. That is really important. I think sometimes that sustainability is just where people go in their next job [with] what they’ve learned….
	With our Youth Advisory Board, we’re providing them the skills, the tools, developing them as leaders. [W]e’re creating a pipeline and saying “*hey*,* these are folks engaged in our work*” [W]e’re hoping that those are also our future employees, or legislators or politicians. They can really speak to, and integrate, this work, but from wherever they want to be. Because [they] could also be a business owner…and say, “*I can use my business to alleviate some of these challenges and concerns*.” [R]eally looking at different ways of involving the community’s [own] initiative into this work. [T]hat makes us a bit different, we’re just like, “*Yeah*,* it’s going to take all of us.”*

The use of italics signals a quote embedded within a quote

## Electronic supplementary material

Below is the link to the electronic supplementary material.

Supplementary Material 1

## Data Availability

UCSD IRB provided a waiver of informed consent to conduct the evaluation. However, this waiver does not include approval to release the qualitative data in its entirety. Access to the datasets generated and analyzed during the current study are restricted to protect the confidentiality of the participants. Reasonable requests for deidentified qualitative datasets can be directed to Todd Gilmer, PhD and are conditional on approval by the Los Angeles County Department of Mental Health.
