# Supplementary material for: An emerging knowledge exchange framework: Leadership insight into a key capacity-building impact in a large urban, trauma-informed initiative supporting resiliency and promoting equity
Source: BMC Public Health. 2025 May 13;25:1746. doi: 10.1186/s12889-025-22268-4 (PMC12070753; doi:10.1186/s12889-025-22268-4)
Supplement: Supplementary file 1 — Supplementary Material 1 [file 12889_2025_22268_MOESM1_ESM.docx]

**Supplemental Materials**

These supplemental materials further contextualize how knowledge exchange took place during the Innovation 2 (INN2) initiative. Quantitative data were collected through the INN2 Health Outcomes Management System (iHOMS) website, a secure data collection and reporting database developed by UCSD Health Services Research Center (HSRC). The system was tailored to support the unique nature of INN2 and was used by agencies as their primary data collection platform for INN2 activities and outcomes. The iHOMS system was launched in August 2018 to support documentation of partnership activities and was updated in 2019 to include the partnership and participant outcome measures. iHOMS’ Event Tracker documented engagement opportunities, meetings and trainings across stakeholders and partnerships.

**Table 1** summarizes opportunities for training in trauma-informed practices among 602 of all partnership members (63%) who completed the assessment, coinciding with agency lead qualitative interviews.. The percentages reported in Table 1 include one response per partnership member within a year. For 2019 only, percentages reported in Tables 1 and 2 represent six months. Opportunities for trauma-informed education and training was widespread: 36% of members reported that their partnership offered training on all 19 trauma-informed practices within the first three years of the initiative, and an additional 33% reported training opportunities on 13-18 practices during the same timeframe. Only 6% of partners reported they had not received training in trauma-informed practices or were unsure if training opportunities were available. Most partners reported that their partnership offered training to impart foundational knowledge related to understanding traumatic stress (84%), how traumatic stress affects the brain and body (82%), and the relationship between mental health and trauma (86%). Fewer partners, though still a strong majority, agreed that their partnership offered training on how to develop crisis and prevention plans (63%).

**Table 1** Partnership-based trauma-informed trainings

| Educational Training Topics | Year 1, 2019*: % of Partnerships Providing Training | Year 2, 2020: % of Partnerships Providing Training | Year 3, 2021: % of Partnerships Providing Training |
| --- | --- | --- | --- |
| What traumatic stress is | 79% | 85% | 86% |
| How traumatic stress affects the brain and body | 75% | 82% | 85% |
| The relationship between mental health and trauma | 75% | 86% | 89% |
| The relationship between substance use and trauma | 62% | 69% | 76% |
| The relationship between homelessness and trauma | 63% | 72% | 73% |
| How trauma affects a child’s development | 75% | 80% | 81% |
| How trauma affects a child’s attachment to his/her caregivers | 62% | 69% | 72% |
| The relationship between childhood trauma and adult re-victimization (e.g., domestic violence, sexual assault) | 63% | 69% | 72% |
| Different cultural issues (different cultural practices, beliefs, rituals) | 64% | 69% | 76% |
| Cultural differences in how people understand and respond to trauma | 60% | 71% | 76% |
| How working with trauma survivors impacts all of us | 69% | 76% | 78% |
| How to help community members identify triggers (e.g., reminders of dangerous or frightening things that have happened in the past) | 60% | 70% | 76% |
| How to help community members manage their feelings (e.g., helplessness, rage, sadness, terror, etc.) | 61% | 72% | 77% |
| De-escalation strategies (i.e., ways to help people to calm down before reaching the point of crisis) | 61% | 69% | 76% |
| How to develop safety and crisis prevention plans | 48% | 61% | 65% |
| How to establish and maintain healthy boundaries | 57% | 75% | 76% |
| How to understand the relationship between poverty and trauma | 57% | 70% | 72% |
| Understanding how poverty and trauma may impact cognitive abilities, behavior and/or engagement (in treatment, school, work etc.) | 60% | 71% | 73% |
| How to develop a common vocabulary to describe the impact of stress and trauma on individuals | 66% | 72% | 77% |
| TOTAL NUMBER OF PARTNERSHIP MEMBERS RESPONDING | 147 | 393 | 383 |

*2019 represents a six-month period, while 2020 and 2021 are full years.

Note: Only one response per partnership member recorded within a year

Within partnerships, capacity-building included partnership meetings and trauma-informed practice educational opportunities for partnership members (Table 1, above). **Table 2** provides a summary of partnership and community capacity-building activities from January 2019 through December 2021. Partnership meetings were used to collaboratively develop programming, problem-solve, and share resources among partners. In 2019, programs were in the early phase of implementation, setting the foundation for the groups and activities, as well as building relationships with partners. In 2020, meetings transitioned to virtual spaces (Zoom) as a result of the COVID-19 pandemic, which resulted in increased meeting attendance. Training opportunities and activities were adapted for virtual spaces, and this continued to be an option in 2021.

Community Partners were asked to keep track of community-capacity building events in the 'Event Tracker' form located in iHOMS. Partners first listed the name of the event being tracked (e.g., Knitting Circles) then classified the event as one of the following options: Outreach and Engagement, Community Event (e.g., Family Fun Night, Health Fair, Outreach Event, Kickoff), Partner Event, Group Activity (e.g., Self-Help Groups, Support Groups, Parenting Groups, Healing Activity Group), Meetings, Training or Educational Workshop, Screening, Resources and Referrals, Hiring, or COVID-19 related. See Figure S1 for examples of how partnership members categorized different community engagement opportunities.

**Figure 1** illustrates the community engagement opportunities that supported knowledge exchange, as tracked in iHOMS. These engagement opportunities fell into four primary areas classified by partnership members: community outreach (e.g., services such as meal delivery, mobile showers), community events (e.g., mindfulness hangouts, resourced drop-in sites for transition age youth), group activities for community members (e.g., knitting and storytelling circles for families with intergenerational trauma) and trainings (e.g., anger management skills, health and safety practices during COVID-19) provided directly to community members. We have qualitatively grouped these four partnership-identified categories by the three key mechanisms, or gears, supporting knowledge exchange, as identified by agency leads and illustrated in Figure 2 of the main text: Creative Partnering, Supportive Frameworks and Trainings, and Skill-building and Application. Any specific activity may overlap among these three mechanisms (e.g., reflect both Creative Partnering and Supportive Framework/Trainings) but only the primary mechanism is identified in Figure 1 to avoid duplication. This figure provides specific examples of community-based engagement activities during the INN2 initiative.

**Table 2** Partnership activities in support of knowledge exchange

| Event Type | Year 1 (2019) | | Year 2 (2020) | | Year 3 (2021) | |
| --- | --- | --- | --- | --- | --- | --- |
|  | # Events | # Attendees | # Events | # Attendees | # Events | #Attendees |
| Partnership Capacity-Building | | | | | | |
| Partnership Meetings | 229 | 1,724 | 681 | 9,373 | 1,075 | 30,315 |
| Partnership Member Trainings | 157 | 1,657 | 868 | 12,303 | 873 | 21,608 |
| Community Capacity-Building | | | | | | |
| Community Outreach | 577 | 13,344 | 2,040 | 72,638 | 2,659 | 222,892 |
| Community Event | 259 | 7,272 | 489 | 30,175 | 1,242 | 125,265 |
| Group Activities in the Community | 253 | 3,472 | 2,020 | 23,962 | 3,821 | 51,787 |
| Community Trainings | 227 | 2,259 | 1,332 | 11,350 | 1,790 | 23,504 |

**Figure 1** Community capacity building opportunities supporting knowledge exchange and addressing community needs
